# Bilateral patellar tendon rupture in a weightlifter during an acute high-loading resistance exercise bout: A case study

**DOI:** 10.17159/2078-516X/2022/v34i1a11781

**Published:** 2022-01-01

**Authors:** LA Alexander, JT Mchunu, RD Kgabu, EW Derman

**Affiliations:** 1 Orthopaedic Registrar, Department of Orthopaedics, Faculty of Health Sciences, School of Clinical Medicine, University of the Witwatersrand, South Africa; 2 Consultant Orthopaedic Surgeon, Department of Orthopaedics, Faculty of Health Sciences, School of Clinical Medicine, University of the Witwatersrand, South Africa; 3 Institute of Sport and Exercise Medicine, Division of Orthopaedic Surgery, Department of Surgical Sciences, Faculty of Medicine and Health Sciences, Stellenbosch University, South Africa; 4 IOC Research Centre, South Africa

**Keywords:** extensor mechanism, load, injury, sport

## Abstract

Bilateral patellar tendon ruptures are exceedingly uncommon, especially when they occur in individuals without predisposing risk factors or systemic disease. Due to its rarity, many cases are missed on initial presentation resulting in poor patient outcomes. Identifying associated risk factors aids in diagnosis and mitigates this oversight. We report a case of a healthy, recreational weightlifter who sustained bilateral patellar tendon ruptures during an acute high-loading resistance exercise bout. We discuss how a spike in acute workload may have predisposed our patient to this injury. Research into training load and athlete injury risk is currently in vogue, however, no studies have analysed whether poor load management increases the risk of tendon ruptures. This case prompts awareness for clinicians who diagnose and manage this injury and helps to stimulate the formation of educational initiatives for athletes and coaches, aimed at injury prevention.

## Case report

Bilateral patellar tendon ruptures are exceedingly uncommon, especially when they occur in individuals without predisposing risk factors or systemic disease. ^[1,2]^ Due to its rarity, many cases are missed on initial presentation resulting in poor patient outcomes.^[1]^ Identifying associated risk factors could potentially mitigate this oversight.^[1]^ Understanding the mechanism of injury and having a high index of suspicion could aid accurate diagnosis.

Rupture typically occurs during a jumping event, when a strong quadriceps contraction is paired with a knee flexed at around 60 degrees.^[3]^ The most frequently reported symptoms include knee pain, immediate knee swelling and difficulty bearing weight. Physical examination may reveal a high-riding patella, a large haemarthrosis, a palpable gap at the inferior pole of the patella, and a patient who is unable to perform a straight leg raise. Treatment of complete tears and/or those with extensor mechanism disruption, is typically surgical repair.^[2]^

We report a case of a healthy, recreational weightlifter who sustained bilateral traumatic patellar tendon ruptures. We discuss how poor load management may have predisposed our patient to this injury.

### History

A 32-year-old male, recreational weightlifter presented with a history of severe, acute, bilateral knee pain and swelling. He reported hearing a sharp popping sound whilst attempting to perform a weighted deep squat manoeuvre the previous day. He subsequently collapsed to the floor and was unable to walk thereafter. He had no known medical comorbidities and no known risk factors associated with tendon ruptures. Specifically, he denied using fluroquinolones, anabolic or corticosteroids, and reported no history of patellar tendinopathy or related injuries.

A sporting history revealed that our patient only had five months of weightlifting experience and that he had significantly and rapidly increased the resistance load on the index deep squat manoeuvre from what he was previously accustomed to. His current one-repetition maximum (1RM) was 42 kg and here a weight of 84 kg was attempted. The patient had previously been training at his home gym but was afforded the opportunity to use heavier weight options at the new training facility located at his place of employment.

### Physical examination

On inspection of both knees, they appeared swollen with superiorly displaced patellae noted. The displacement and effusions were confirmed on palpation. Furthermore, his knees were tender on palpation mainly over the inferior patellae poles. He was unable to perform a straight leg raise or actively extend either knee, confirming disrupted lower limb extensor mechanisms. Standard clinical tests for ligament and meniscal injury were unremarkable. There was no neurovascular compromise. Initial differential diagnosis included bilateral patella fractures, quadriceps tendon injuries and patellar tendon injuries.

### Imaging and diagnosis

**Figs 1a/1b:**
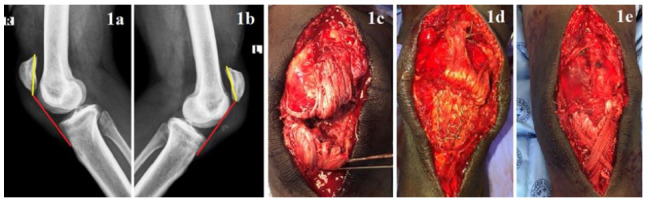
Lateral knee X-Rays showed increased Insall-Salvati Index (Left: 2.0, Right: 2.1).^[1]^ **Fig. 1c.** Intra-op findings of a patellar tendon rupture occurring at the inferior pole, with damage to the retinaculum. **Fig. 1d.** Left and right patellar tendons were repaired surgically using non-absorbable suture Krakow stiches. **Fig. 1e.** The repair was re-enforced with non-absorbable mesh tape arranged in a figure-of-eight pattern around the circumference of the patella and through a horizontal drill-hole in the tibial tubercle.

X-rays revealed an increased Insall-Salvati Index on both sides (Left: 2.0, Right: 2.1) with bony avulsion, inferior to the left patella, most likely arising from the inferior pole (Fig. 1a/1b).^[1]^ A final diagnosis of bilateral patellar tendon ruptures was made. The patient was admitted to hospital, where his lower extremities were immobilised in full extension. Analgesic drugs were administered and he was prepped for surgical intervention.

### Surgery and its outcome

Intra-operative findings confirmed bilateral patellar tendon ruptures arising from the inferior patella poles, with an associated bony avulsion on the left (Fig. 1c).

Acute tears are treated surgically via direct primary repair. End-to-end repairs are used for acute mid-substance tears and transosseous sutures or suture anchors are used for proximal or distal injuries. This differs from chronic tears, where reconstruction using an autograft or allograft is preferred. ^[3]^

Our definitive management involved a traditional, direct primary surgical repair with non-absorbable transosseous Krakow whip sutures augmented with a surrounding non-absorbable mesh tape (Fig. 1d). The retinaculum was repaired bilaterally (Fig. 1e). No surgical complications resulted.

After follow-ups at two, six and 12 weeks, whilst adhering to a comprehensive multidisciplinary rehabilitation plan, the patient made a full recovery. Contact at six months revealed full range of motion in both knees and a successful return to his pre-injury sporting level.

## Discussion

The patellar tendon forms part of the knee extensor mechanism and consists of tightly packed parallel collagen fibres capable of significant tensile strength, such that acute rupture of a healthy tendon is extremely uncommon.^[3]^ Most ruptures occur at the inferior pole followed by the mid-substance area.^[3]^ Unilateral extensor mechanism injuries are 15 times more common than bilateral injuries.^[2]^

With regards to weight-bearing structures, an Achilles tendon rupture is far more common than a patellar tendon rupture. The low incidence is a result of the bony patella, considered the weakest part of the extensor mechanism, failing before the adjacent tendinous components.^[4]^

Patellar tendon rupture cases are distributed bimodally. Seventy percent are seen in the seventh decade of life and are mostly related to spontaneous ruptures associated with chronic medical conditions (e.g. renal disease or systemic lupus erythematosus) or medication use (e.g. fluroquinolones).^[1,2]^ The remainder of cases are seen in individuals under the age of 40 during sports such as netball and weightlifting. In athletes, tendon rupture is usually the result of long-standing chronic tendon degeneration due to repetitive microtrauma and subsequent tendinopathy.^[1]^

Other athletes at risk are those using anabolic agents or corticosteroids. However, due to these agents being banned in sport and subsequent non-disclosure, their true incidence related to tendon rupture is unknown. Despite specific enquiry, none of these risk factors were demonstrated in our patient. Notably, our patient was very inexperienced and had never received guidance on how to formulate a safe training programme or how to complete these movements with correct techniques and form. Our patient’s index repetition was 110% heavier than his 1-RM which was a substantial and rapid increase in resistance, ultimately resulting in his injury.

The principle of tissue overload is widely practiced in sport and involves the application of gradually increasing stress over time to the body, over and above that which is normally encountered, to elicit physiological adaptation and subsequent performance improvement. Furthermore, load management is increasingly being recognised for its fundamental role in injury prevention.^[5]^ Gabbett et al. found that if weekly training load was increased by more than 15% in some athletes, their injury risk may increase by up to 49%. Therefore, they recommend that athletes should limit weekly training load increases to <10%.^[5]^ Rabello et al. demonstrated a relationship between increases in weekly training load and a participant’s patellar tendon structure using ultrasound tissue characterisation techniques. They concluded that these structural changes may correlate with an increased risk of tendon-related injuries.^[6]^ These studies support our belief that the acute rate of significant load increase played a pivotal role in our patient sustaining his injuries.

## Conclusion

Patellar tendon ruptures are uncommon, and bilateral concurrent pathology is considered extremely rare.^[2]^ This case accompanies a select few reports which detail a sports-related bilateral patellar tendon rupture.^[1,3]^ Furthermore, despite the increasing popularity in sports using muscle power, there have been no published reports of this injury in amateur or professional weightlifters.

When clinicians are confronted with an amateur athlete, a thorough sporting history may help in identifying a potential risk factor, such as poor load management and assist in confirming this rare diagnosis. Athletes should be counselled on how anabolic agents, corticosteroids and fluroquinolones increase one’s risk of tendon rupture. Educational initiatives which focus on safe training practices for injury prevention should be formulated.

This report highlights the paucity of literature available on bilateral patellar tendon ruptures during acute high-loading resistance exercise and it provides a framework for future research into load management and tendon injuries. The report also creates awareness for orthopaedic surgeons, emergency medicine physicians and sports physicians who diagnose and manage these injuries.

## References

[1] Camarda L Arienzo AD Morello S Bilateral ruptures of the extensor mechanism of the knee: A systematic review J Orthop 2017 14 14 445 453 10.1016/j.jor.2017.07.008 28819342 PMC5548366

[2] Monroy A Urruela A Egol KA Bilateral disruption of soft tissue extensor mechanism of knee: Functional outcome and comparison to unilateral injuries HSS J 2013 9 1 12 16 10.1007/s11420-012-9314-8 24426838 PMC3640716

[3] Pengas IP Assiotis A Khan W Adult native knee extensor mechanism ruptures Injury 2016 47 10 2065 2070 10.1016/j.injury.2016.06.032 27423309

[4] Clayton RA Court-Brown CM The epidemiology of musculoskeletal tendinous and ligamentous injuries Injury 2008 39 12 1338 1344 10.1016/j.injury.2008.06.021 19036362

[5] Hulin BJ Gabbett TJ Lawson DW The acute:chronic workload ratio predicts injury: high chronic workload may decrease injury risk in elite rugby league players Br J Sports Med 2016 50 4 231 236 10.1136/bjsports-2015-094817 26511006

[6] Rabello LM Zwerver J Stewart RE Patellar tendon structure responds to load over a 7 - week preseason in elite male volleyball players Scand J Sci Sport 2019 29 7 992 999 10.1111/sms.13428 30942914 PMC6850050

